# Sperm Proteases that May Be Involved in the Initiation of Sperm Motility in the Newt, *Cynops pyrrhogaster*

**DOI:** 10.3390/ijms150915210

**Published:** 2014-08-28

**Authors:** Misato Yokoe, Makoto Sano, Honami Shibata, Daisuke Shibata, Eriko Takayama-Watanabe, Kazuo Inaba, Akihiko Watanabe

**Affiliations:** 1 Department of Biology, Faculty of Science, Yamagata University, 1-4-12 Kojirakawa, Yamagata 990-8560, Japan; E-Mails: yokoe@sbiol.kj.yamagata-u.ac.jp (M.Y.); sano@sbiol.kj.yamagata-u.ac.jp (M.S.); shibata@sbiol.sci.kj.yamagata-u.ac.jp (H.S.); 2 Shimoda Marine Research Center, University of Tsukuba, 5-10-1 Shimoda, Shizuoka 415-0025, Japan; E-Mails: shibata@kurofune.shimoda.tsukuba.ac.jp (D.S.); kinaba@kurofune.shimoda.tsukuba.ac.jp (K.I.); 3 Institute of Arts and Sciences, Yamagata University, 1-4-12 Kojirakawa, Yamagata 990-8560, Japan; E-Mail: ewatanabe@kdw.kj.yamagata-u.ac.jp

**Keywords:** acrosome reaction, sperm motility, serine protease, acrosins, 20S proteasome, cysteine protease, AEBSF (4-(2-aminoethyl) benzenesulfonyl fluoride), urodele

## Abstract

A protease of sperm in the newt *Cynops pyrrhogaster* that is released after the acrosome reaction (AR) is proposed to lyse the sheet structure on the outer surface of egg jelly and release sperm motility-initiating substance (SMIS). Here, we found that protease activity in the sperm head was potent to widely digest substrates beneath the sperm. The protease activity measured by fluorescein thiocarbamoyl-casein digestion was detected in the supernatant of the sperm after the AR and the activity was inhibited by 4-(2-aminoethyl) benzenesulfonyl fluoride (AEBSF), an inhibitor for serine or cysteine protease, suggesting the release of serine and/or cysteine proteases by AR. In an *in silico* analysis of the testes, acrosins and 20S proteasome were identified as possible candidates of the acrosomal proteases. We also detected another AEBSF-sensitive protease activity on the sperm surface. Fluorescence staining with AlexaFluor 488-labeled AEBSF revealed a cysteine protease in the principal piece; it is localized in the joint region between the axial rod and undulating membrane, which includes an axoneme and produces powerful undulation of the membrane for forward sperm motility. These results indicate that AEBSF-sensitive proteases in the acrosome and principal piece may participate in the initiation of sperm motility on the surface of egg jelly.

## 1. Introduction

In the internal fertilization of urodeles, quiescent sperm stored by females are inseminated in the egg jelly matrix around an ovulated egg and then initiate motility [1,2]. We previously identified a 34-kDa protein as a sperm motility-initiating substance (SMIS) in the newt, *Cynops pyrrhogaster* [3]. The SMIS is localized in the granules near the outer surface of egg jelly, where quiescent sperm are mechanically inseminated at the beginning of natural fertilization. The granules are sequestered from outside by the sheet structure where an acrosome reaction-inducing substance (ARIS) is localized [3]. Based on the unique localizations of SMIS and ARIS, we proposed a new role of the acrosome reaction (AR) in the initiation of sperm motility at fertilization; acrosomal proteases would act on the sheet structure and release SMIS as a proteolytic product [3].

In the sperm AR, exocytosis of the acrosomal vesicle exposes multiple acrosomal enzymes around the sperm surface [4,5,6,7]. Serine proteases are known as the major acrosomal enzymes in many animal species such as mammals [8,9], Aves [10], ascidians [7], and echinoderms [11,12]. They are thought to be necessary for sperm penetration into the zona pellucida and vitelline envelope, because fertilization is blocked by various protease inhibitors [13,14]. For example, 26S and 20S proteasomes exposed by the AR are responsible for the lysis of egg coat in ascidian and avian sperm [15,16]. However, some serine proteases exposed by the acrosomal exocytosis are not essential for digestion of substrates in egg coat [17,18] and have another role in dispersal of acrosomal proteins [19]. These indicate that the acrosomal proteases have multiple roles in fertilization in animal species.

In the newt *C. pyrrhogaster*, the role of acrosomal protease in the steps of sperm-egg interaction is unclear, although a protease sensitive to soybean trypsin inhibitor is shown to be present in the tip of the sperm head [20]. In the present study, we examined the localization and characterization of sperm protease that participates in the initiation of sperm motility during the internal fertilization of *C. pyrrhogaster*. We found that a protease sensitive to an inhibitor for serine and papain-like cysteine proteases, 4-(2-aminoethyl) benzenesulfonyl fluoride (AEBSF) [21], was released by the AR. Sperm-specific serine proteases, acrosins and 20S proteasome were detected as possible candidates for the acrosomal proteases in *in silico* analysis. We also found that another papain-like cysteine protease is present in the principal piece. These AEBSF-sensitive sperm proteases are suggested to be involved in the initiation of sperm motility.

## 2. Results and Discussion

### 2.1. Acrosomal Protease in C. pyrrhogaster Sperm

**Figure 1:**
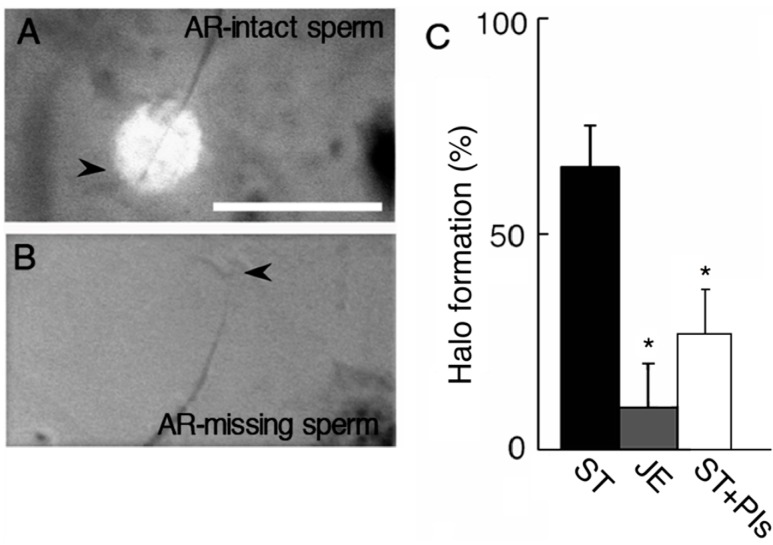
Digestion of gelatin film by sperm acrosomal protease. Sperm were put on a gelatin-coated glass slide and then air-dried. Gelatin was stained with Coomasie Brilliant Blue R-250. (**A**) An acrosome-intact sperm. A halo of gelatin digestion was observed as an unstained area around the sperm acrosome; (**B**) An acrosome-reacted sperm. No gelatin digestion was observed. Arrows indicate tip of sperm head. Bar: 25 µm; and (**C**) Decrease in percentages of the sperm with a halo of gelatin digestion by treatment with JE. * *p* < 0.01 against that in acrosome-intact sperm (Intact). ST: modified Steinberg’s salt solution, JE: egg jelly extract, PIs: protease inhibitor cocktail, AR: acrosome reaction.

We determined the localization of protease activity in *C. pyrrhogaster* sperm by evaluating the gelatin digestion in a thin gelatin film. Gelatin was stained with Coomasie Brilliant Blue (CBB) on the glass slide and the digestion was observed as a CBB-unstained halo around the tip of the sperm head. The halo was typically formed around acrosome-intact sperm (Figure 1A), whereas it was rarely observed around acrosome-missing sperm (Figure 1B). No digestion was reproducibly observed in the other area beneath the sperm. The halo formation was rarely observed in the sperm pretreated with protease inhibitor cocktail or just after the treatment of sperm with egg jelly extract (JE) (Figure 1C), which contains AR-inducing activity [22]. This suggests that proteases which lyse the substrates around sperm are present only in the acrosome and that most of the acrosomal proteases are diffused by AR.

**Figure 2:**
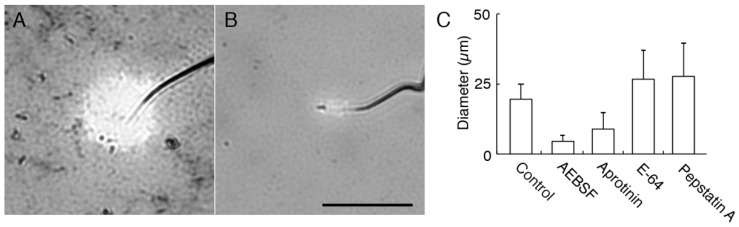
Inhibitory effects of protease inhibitors on halo formation. One of the four kinds of protease inhibitors—AEBSF (4-(2-aminoethyl) benzenesulfonyl fluoride), aprotinin, E-64, and pepstatin A—were added to sperm suspension and smeared on gelatin film. Diameter of halos around the tip of sperm head was measured. (**A**,**B**): Halos around sperm without treating with any inhibitors as control (**A**) and AEBSF-treated sperm (**B**). Bar: 25 µm; (**C**) Diameters of the halo around sperm in treatment with protease inhibitors.

To characterize the acrosomal proteases released by AR, effects of halo formation on AEBSF, and three kinds of inhibitors with specificity to serine and cysteine proteases, cysteine proteases, and asparate proteases; aprotinin, E-64 and pepstatin A, respectively, were examined. The inhibitory effects on halo formation were apparent in AEBSF and aprotinin; diameters of halos were reduced (Figure 2). The inhibitory effect was severely detected especially in AEBSF.

**Figure 3:**
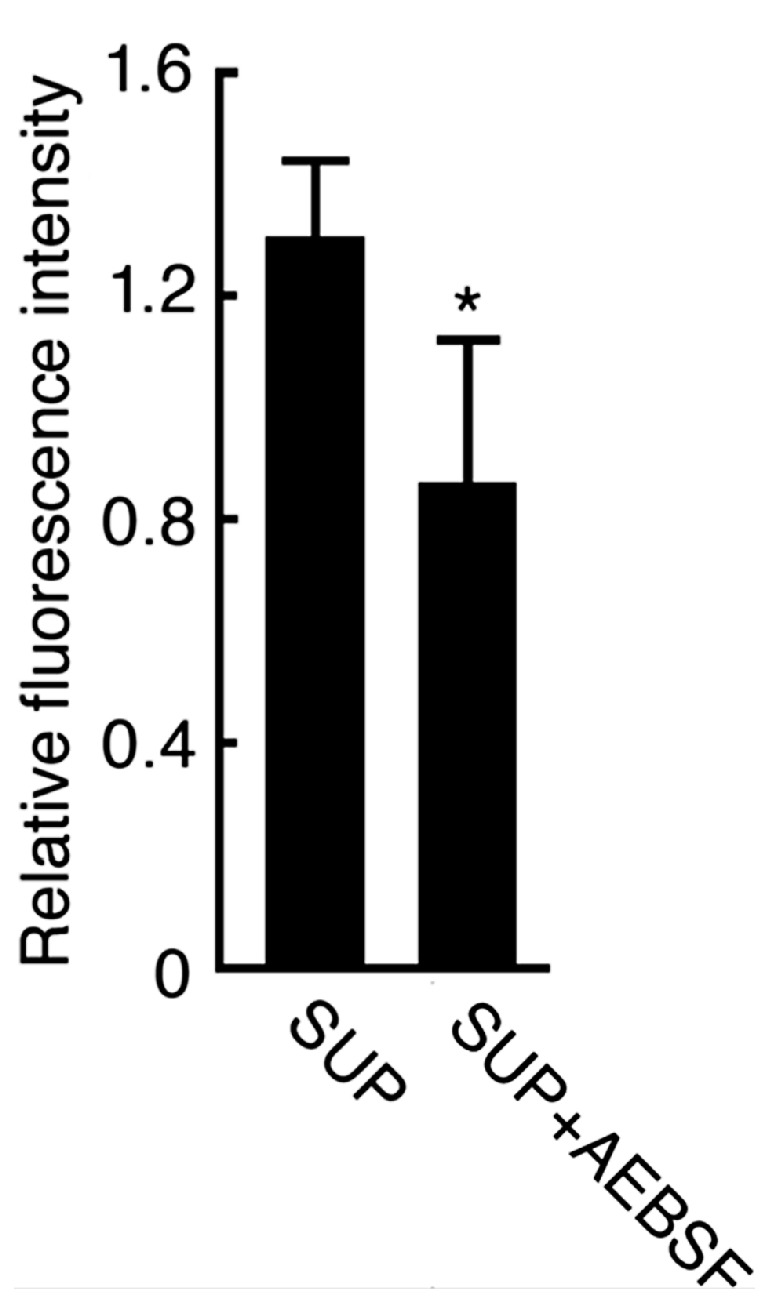
Detection of AEBSF-sensitive protease activities in the supernatant of the AR-induced sperm. AR was induced in 50 µg/mL DBA, and the supernatant (SUP) was treated with FTC (fluorescein thiocarbamoyl)-casein or co-treated with FTC-casein and AEBSF for 1 h. Sperm treated with 50 µg/mL DBA (*Dolichos biflorus* agglutinin) and 10 mg/mL GalNAc (*N*-acetylgalactosamine) were used as the control. Fluorescence intensities of those solutions were measured by a fluorometer. Relative fluorescence intensities were calculated by the ratio against the fluorescence intensity of the supernatant of the sperm whose acrosome reaction was inhibited by GalNAc. * *p* < 0.01 compared to the relative fluorescence intensity from FTC-casein reacted with supernatant of DBA-treated sperm.

Alternatively, we suspended sperm in JE and analyzed the supernatant by fluorescein thiocarbamoyl (FTC)-casein assay, in which proteolytic activity is measured by the increase in fluorescence. When AR was induced by JE, no reproducible result was obtained, probably because of interference by crude contents. Therefore, we used *Dolichos biflorus* agglutinin (DBA), which is shown to artificially induce AR in sperm of *C. pyrrhogaster* [23]. The fluorescence intensity in the supernatant of the AR-induced sperm was 1.3 ± 0.14-fold higher than that of the supernatant of the sperm whose AR was inhibited by GalNAc (*N*-acetylgalactosamine) (Figure 3) [23]. AEBSF inhibited the protease activity in the supernatant of the AR-induced sperm; the fluorescence intensity was 0.86 ± 0.25-fold higher than that of the supernatant of the sperm whose AR was inhibited. The relative fluorescence intensity of the supernatant was significantly low in the presence of AEBSF (*p* < 0.01). Form these results, we concluded that most acrosomal proteases released by the AR are serine or cysteine proteases.

In *C. pyrrhogaster*, AR is induced in inseminated sperm on the sheet structure that covers the outer surface of the egg jelly and sequesters granules containing SMIS [3,22]. Significance of AR in the outer surface of the egg jelly was demonstrated by the report that the fertilization rate is decreased when AR-induced sperm are inseminated to the intact egg [24]. That decrease of the fertilization rate is ascribed to the dysfunction of the AEBSF-sensitive acrosomal protease on the surface of egg jelly [24]. At the beginning of *Cynops* fertilization, digestion of sheet structure on the surface of egg jelly results in the exposure of granules containing SMIS [25], which induces the initiation of sperm motility [1]. This process is significant for sperm to penetrate jelly matrix with enhanced motility [26]. The AEBSF-sensitive acrosomal protease is a strong candidate for the digestion of sheet structure and may act as a critical trigger for the interaction of sperm with the egg jelly surface.

### 2.2. Effect of AEBSF (4-(2-Aminoethyl) benzenesulfonyl fluoride)-Sensitive Acrosomal Protease in AR (Acrosome Reaction)-Induced Sperm

**Figure 4:**
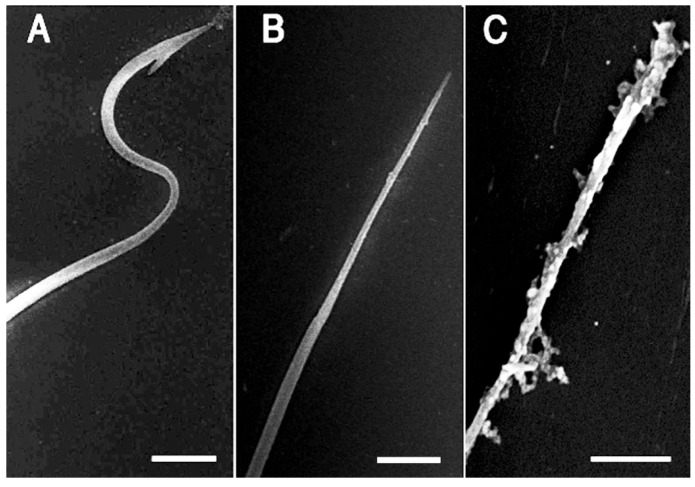
Scanning electron microscopic observation of the AR-induced sperm in the JE containing AEBSF. Sperm were incubated in ST, JE, or JE containing 1 mg/mL AEBSF for 5 min, and then fixed in 2.5% glutaraldehyde. Tip of the sperm head was observed by scanning electron microscope. (**A**–**C**) indicate intact acrosome (**A**), exposed perforatorium (**B**), and perforatorium with much debris (**C**) that were typically observed at a tip of sperm head in the ST, the JE, and the AEBSF-containing JE, respectively. Bars: 1 µm.

The AEBSF-sensitive acrosomal proteases are thought to be released to catalyze the substrate around sperm in *C. pyrrhogaster*, whereas, in mice, one of the acrosomal serine proteases, acrosin is reported to play a role to disperse acrosomal proteins [19]. To know the effect of the acrosomal proteases on the acrosome reacted sperm themselves, we observed sperm treated with JE in the absence or presence of AEBSF by scanning electron microscopy. Acrosome of sperm in a physiological salt solution (ST: modified Steinberg’s salt solution) mostly appeared intact (98.0% ± 1.2%) (Figure 4A). Sperm in the JE showed exposure of an internal acrosomal structure, the perforatorium (99.4% ± 0.6%) (Figure 4B). Sperm treated with AEBSF-containing JE also lost the acrosome (93.3% ± 0.5%). In this case, all sperm exhibited perforatorium with much debris on their surface (Figure 4C). This result indicates that acrosome contains an AEBSF-sensitive protease responsible for complete exposure of perforatorium in the AR-induced sperm.

At the internal fertilization of *C. pyrrhogaster*, AR-induced sperm initiate appropriate motility on the egg jelly surface and begin to penetrate into egg jelly [27]. The gelatinous nature of the jelly matrix provides a mechanical guide for the sperm to the surface of the vitelline envelope [28], whereas it excludes abnormal sperm in morphology or motility by being trapped in the matrix [22]. The complete exposure of perforatorium is thought to make AR-induced sperm efficiently penetrate through the jelly matrix without being trapped by it.

### 2.3. In Silico Detection of Acrosins and 20S Proteasomes Possibly Expressed in Sperm

**Figure 5:**
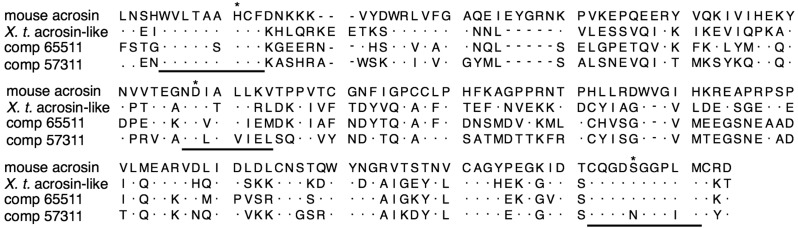
Sequence alignment of amino acid in catalytic domain of acrosins. Catalytic domains of mouse acrosin and acrosin-related protein of *Xenopus tropicalis* (*X. t.*) were obtained from NCBI gene database (NIH, Bethesda, MD, USA). Deduced amino acid sequences of comp 65511 and 57311 [31] were aligned on CLC workbench platform. Underlines were conserved regions of serine proteases for their proteolytic activity. Asterisks indicate strongly conserved amino acid residues in the catalytic domain.

**Figure 6:**
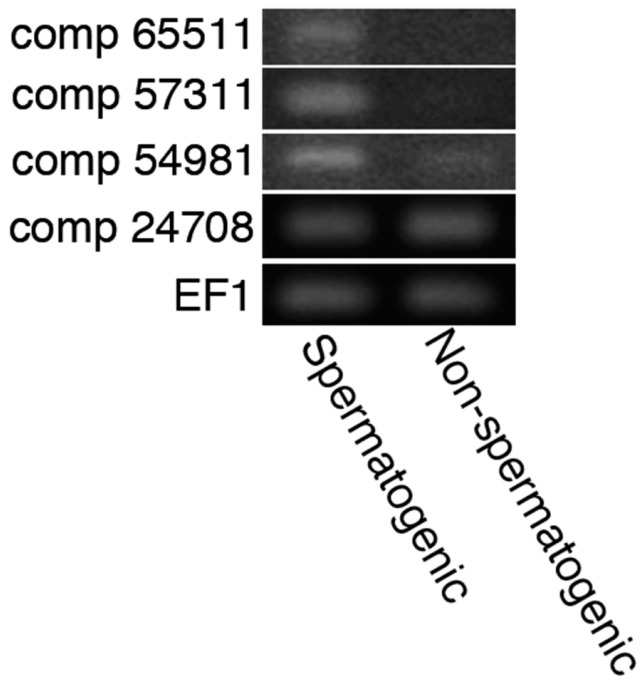
Expressions of the genes with contig DNA sequences in spermatogenic or non-spermatogenic testes. RT-PCR was performed using 1 µg of total RNA purified from spermatogenic or non-spermatogenic testes. Details are in Experimental section.

Multiple roles of acrosomal protease in the fertilization of animal species are ascribed to distinct proteases such as acrosin and proteasomes [15,16,19]. In order to examine whether those proteases were also present in the sperm of *C. pyrrhogaster*, we performed RNA-seq of the testes. In the data of contig DNAs produced by *de novo* transcriptome assembly, we found two base sequences encoding proteins with high homology to mouse acrosin (accession: AAA371631); comp 65511 (score: 323, E-value: 9 × 10^−104^) and 57311 (score: 301, E-value: 1 × 10^−94^). The deduced amino acid sequences were constructed by 370 (comp 65511) and 434 (comp 57311) amino acids, both of which included the catalytic domain conserved in serine proteases (Figure 5) [29]. Transcripts including a sequence of each contig DNA were detected in the spermatogenic testes by reverse transcription polymerase chain reaction (RT-PCR), but they were not in the testes that spermatogenesis had been ceasing by keeping at 4 °C (Figure 6). In addition, we found two base sequences encoding proteasomes alpha type 7 and 1 with high homology to 20S proteasome of *Halocynthia roretzi* (accession BAK53482) [30]; comp 54981 (score: 446 and E-value: 3 × 10^−156^) and 24708 (score: 519 and E-value: 0.0). Transcripts including a sequence of comp 54981 were detected strongly in the spermatogenic testes (Figure 6). These results suggest that the sperm of *C. pyrrhogaster* possess at least two kinds of acrosins and possibly one 20S proteasome that may be responsible for multiple roles of acrosomal proteases in fertilization.

### 2.4. Detection and Visualization of Sperm Surface Protease

**Figure 7:**
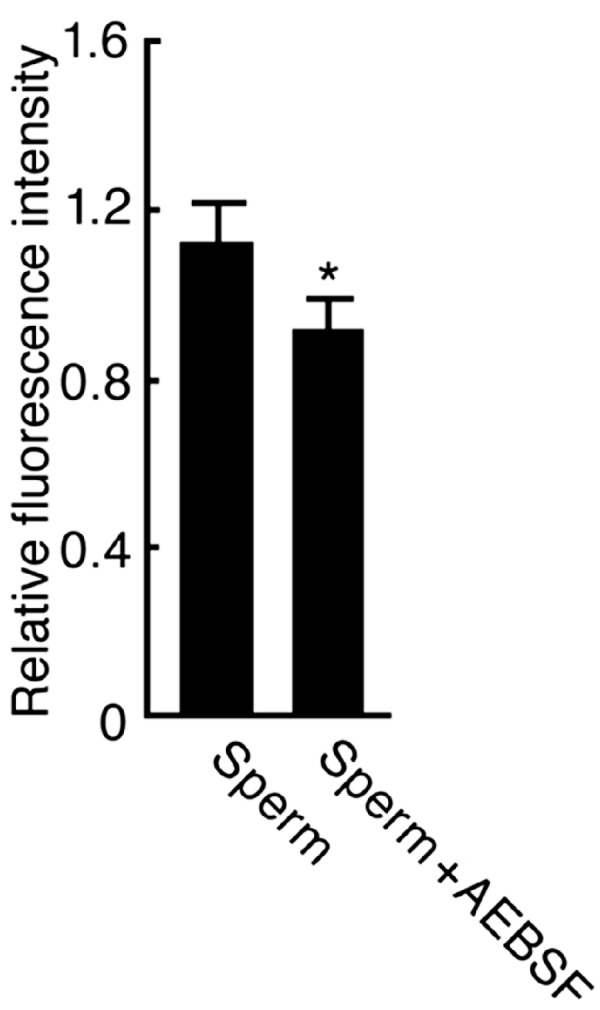
Detection of AEBSF-sensitive protease activity on the sperm surface. Sperm was treated with FTC-casein or co-treated with FTC-casein and AEBSF for 1 h. As the control, FTC-casein alone was incubated for 1 h, and then sperm was added. Fluorescence intensities in the sperm suspensions were measured by a fluorometer. Relative fluorescence intensities were calculated by the ratio against the fluorescence intensity in control sperm suspension including FTC-casein without being co-incubated with sperm. * *p* < 0.01 compared to the relative fluorescence intensity in the control.

In order to further examine the presence of protease activity that localizes on the sperm surface and may contribute to initiate sperm motility, we incubated intact sperm with FTC-casein. The fluorescence intensity from the FTC-casein in the sperm suspension was 1.1 ± 0.092-fold higher than that without incubation with sperm (Figure 7). The FTC-casein degrading activity was suppressed to 0.92 ± 0.074-fold in the presence of AEBSF. The relative fluorescence intensity in the AEBSF-containing sperm suspension was significantly low (*p* < 0.01), suggesting that an AEBSF-sensitive protease is present on the sperm surface.

**Figure 8:**
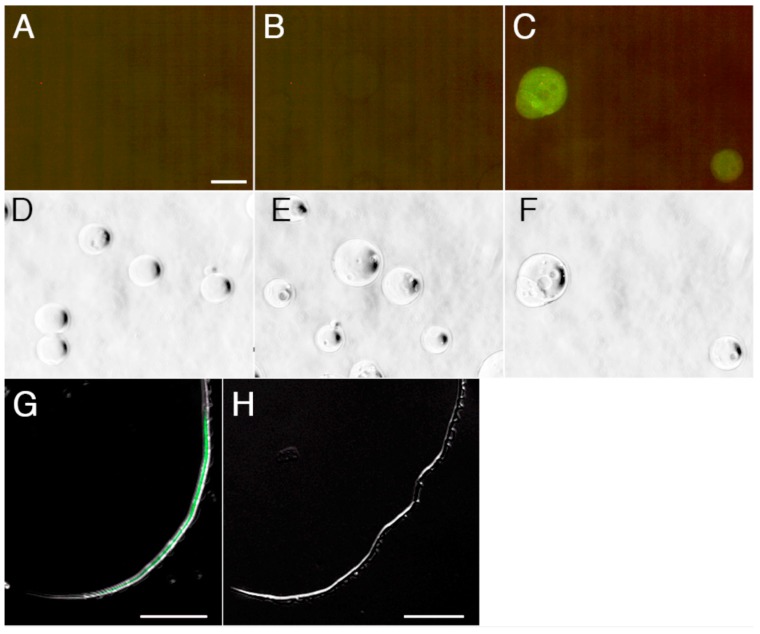
Binding specificity of Alexafluor 488-labeled AEBSF for serine and cysteine proteases. Alexafluor 488-labeled AEBSF was prepared by a covalent binding of Alexafluor 488 to the amino group of AEBSF. (**A**–**F**) Alexafluor 488-labeled AEBSF was treated with trypsin-conjugated agarose beads (**A**,**D**), chymotrypsin-conjugated agarose beads (**B**,**E**), or papain-conjugated agarose beads (**C**,**F**). (**D**–**F**) Bright field of panels (**A**–**C**), respectively; (**G**,**H**) Merged images of dark field and fluorescence of principal piece of the sperm treated with Alexafluor 488-labeled AEBSF (**G**) or co-treated with Alexafluor 488-labeled AEBSF and papain (**H**). Bar: 25 µm.

**Figure 9:**
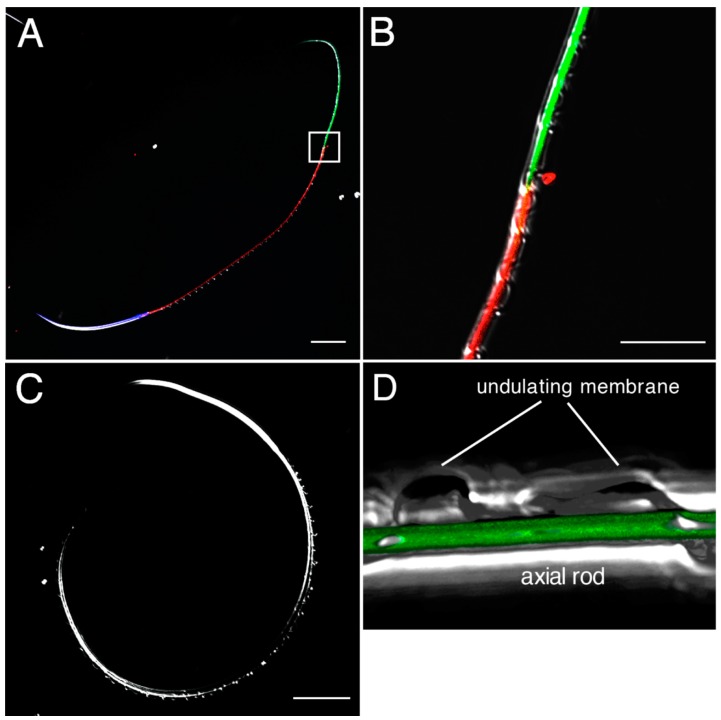
Confocal laser scanning microscopy observations of the Alexafluor 488-labeled AEBSF-bound sperm. (**A**) Sperm was treated with Alexafluor 488-labeled AEBSF, Hoechst 33342 and MitoTracker orange. Green fluorescence from Alexafluor 488 was observed in the principal piece. Blue and red fluorescence derived from Hoechst 33342 and MitoTracker orange, respectively, showing the localizations of the nucleus and mitochondrion; (**B**) High-magnification view of box in (**A**) showing the border between the midpiece and the principal piece. Alexafluor 488-labeled AEBSF bound from the anterior end of the principal piece; (**C**) Sperm not treated with the fluorescence dyes as the control for panel (**A**); and (**D**) 3D-reconstruction of fluorescence images of the principal piece. Alexafluor 488-labeled AEBSF bound to the joint region between axial rod and undulating membrane.

To visualize the localization of AEBSF-sensitive protease on the sperm surface, we prepared Alexafluor 488-labeled AEBSF. An assay using agarose beads conjugated with several proteases indicated that covalent binding of Alexafluor 488 to AEBSF caused loss of the binding specificity to serine proteases (Figure 8A–F). When Alexafluor 488-labeled AEBSF was mixed with the sperm, fluorescence from Alexafluor 488 was observed in the joint region between axial rod and undulating membrane in the principal piece that was recognized by the absence of mitochondrion in the sperm tail (Figure 8G and Figure 9). This region contains an axoneme and is thought to produce undulation of the membrane for forward sperm motility. The fluorescence in the principal piece was not observed by co-treatment of the Alexafluor 488-labeled AEBSF with 1 mg/mL papain (Figure 8H), suggesting that a papain-like cysteine protease is localized in the sperm principal piece. Although it is unclear whether this protease contributes to the digestion of the sheet structure, its localization close to the undulating membrane suggests a possible involvement of the cysteine protease in controlling sperm motility. Egg jelly contains a large amount of inactive SMIS with binding to other jelly substances [3]. The inactivated SMIS is possibly activated by proteolysis with a reduction of its molecular size [3,32]. The cysteine protease in the principal piece may catalyze the cleavage of the SMIS-bound jelly substance or the SMIS itself for producing the active form of SMIS, although the precise mechanism for activation of SMIS at fertilization is not yet clear.

## 3. Experimental Section

### 3.1. Gametes

Mature newts of *C. pyrrhogaster* were captured in Yamagata prefecture, Japan. Ovulation was induced in sexually mature females by administering two injections of gonadotropin (150 IU (International Unit)) (Asuka Pharmacy, Tokyo, Japan) at a 24-h interval. Eggs were obtained from the posterior portion of the oviduct termed the uterus at 2–3 days after the last injection. Sperm were obtained from the vas deferens.

### 3.2. Solutions

Reconstructed ionic solution (RIS) was prepared based on the estimated concentrations of major cations in the egg jelly of *C. pyrrhogaster* [28]. The RIS contained 20 mM NaCl, 2.7 mM KCl, 0.39 mM MgCl_2_, 5.1 mM CaCl_2_, 40 mM choline-Cl, and 10 mM Tris-HCl (pH 8.5). Modified Steinberg’s salt solution (ST) was prepared as 58.2 mM NaCl, 0.67 mM KCl, 6 mM CaNO_3_, 0.83 mM MgSO_4_, and 10 mM Tris-HCl (pH 8.5). Reconstructed vas deferens solution (RVDS) was prepared according to the estimated concentrations of major cations and pH in lumen of the vas deferens as 4 mM NaCl, 1.3 mM KCl, 0.1 mM CaCl_2_, 0.06 mM MgSO_4_, and 10 mM Hepes-NaOH (pH 6.9) [25].

### 3.3. Preparation of JE (Egg Jelly Extract)

Mature eggs were immersed in the RIS or the ST with a volume of 50 µL per egg. Following agitation of the egg suspension at 4 °C for 1 h, the saline was collected and centrifuged at 16,000× *g*, 4 °C for 30 min. The supernatant was the JE, and it was stored at −30 °C.

### 3.4. Detection of Protease Activity

Protease activity was estimated by two different methods. One was the detection of gelatin film digestion previously demonstrated in [20] with some modifications. Briefly, sperm were treated with ST, JE, or ST, including protease inhibitor cocktail (GE Healthcare, Little Chalfont, UK) for 10 min. Inhibitions of the gelatin digestion were also examined using 1 mg/mL AEBSF (Sigma-Aldrich, St. Louis, MO, USA), 2 µg/mL aprotinin (Sigma-Aldrich), 10 µg/mL E-64 (Sigma-Aldrich), or 1 µg/mL pepstatin A (Sigma-Aldrich). They were closely attached onto a gelatin-coated glass slide and air-dried. They were stained with CBB-R250. Gelatin digestion around the sperm was detected as an unstained area. The other was the detection of FTC-casein digestion (ThermoScientific, Rockford, IL, USA). Dry sperm (1 µL) was rinsed with RIS, and 1/100 of the sperm were suspended in 100 µL of the JE or the RIS containing 50 µg/mL *Dolichos biflorus* agglutinin (DBA), which artificially induces the AR in sperm [23]. In the control for DBA action, sperm were co-treated with 50 µg/mL DBA and 50 µg/mL *N*-acetylgalactosamine (GalNAc), a monosaccharide that binds specifically to DBA. The sperm suspension was centrifuged at 2500× *g* for 5 min, and then 50 µL of the supernatant was mixed with an equal volume of 0.32 µg/mL FTC-casein, or with an equal volume of 0.32 µg/mL FTC-casein and 1 mg/mL AEBSF. The mixture was incubated at room temperature for 1 h. For the estimation of the activity of the sperm surface protease, we suspended 1/100 of the rinsed sperm in 50 µL of RIS or RIS containing 1 mg/mL AEBSF. After incubation for 30 min at room temperature, the sperm suspension was mixed with an equal volume of 0.32 µg/mL FTC-casein. The mixture was incubated at room temperature for 1 h. In the control, RIS without sperm was incubated with an equal volume of 0.32 µg/mL FTC-casein for 1 h, and then sperm were added. Fluorescence intensity was measured using a FluoroMax3 (Horiba, Fukuoka, Japan) with the wavelengths 325 nm (excitation) and 512 nm (emission). The ratio of fluorescence intensities in the experimental sample against that in the control sample was calculated as the relative fluorescence intensities. Experiments were performed at least 3 times and statistical differences were estimated using Student’s *t*-test.

### 3.5. Scanning Electron Microscopy

Sperm were suspended in the JE, JE containing 1 mg/mL AEBSF, or the ST for 5 min and fixed with 2.5% glutaraldehyde in 0.1 M phosphate buffer (pH 7.0) at 4 °C overnight. They were rinsed with phosphate buffer, dehydrated and dried in a critical point apparatus (HCP-1; Hitachi, Tokyo). Finally, the sperm were coated with platinum using a magnetron sputter (JUC-5000; JEOL, Tokyo, Japan) and observed with a scanning electron microscope (JSM-6510LV, JEOL).

### 3.6. RNA-seq and De Novo Transcriptome Assembly

RNA-seq data by *de novo* transcriptome assembly of complement DNA from testes was constructed with the large aid of Dr. F. Toyama of Utsunomiya University and Dr. C. Chiba of University of Tsukuba [33]. Total RNA was purified from the spermatogenic testes by Trizol reagent (Life Technologies, Tokyo, Japan) and evaluated with an Agilent 2100 Bioanalyzer (Agilent Technology, Santa Clara, CA, USA). A normalized cDNA library was constructed with a TrueSeq RNA Sample Prep Kit (Illumina, San Diego, CA, USA) followed by Duplex Specific Nuclease normalization (Illumina) according to the manufacturer’s instructions. Paired end sequencing was carried out by Illumina HiSeq2000. The reported sequence data have been deposited in the Sequence Read Archive (SRA) at NCBI (SRP034152). *De novo* transcriptome assembly was performed using Trinity [34].

### 3.7. RT-PCR

Mature males were dissected to obtain spermatogenic testes or non-spermatogenic testes that were prepared by 1-year storage of males at 4 °C before dissection. The storage at low temperature is reported to prevent spermatogenic cells entering into meiosis and results in complete cessation of spermatogenesis [35]. One µg of total RNA purified from spermatogenic or non-spermatogenic testes was transcribed with SMARTScribe reverse transcriptase (Clontech, Mountain View, CA, USA). Polymerase chain reaction was performed using 1 µL of the reacted solution with DNA primers specific for each contig DNA sequences (Table S1). As the control, primers for elongation factor 1 gene (EF1: accession AB005588) were used. Reactions of 30 cycles were carried out with denature at 95 °C for 30 s, annealing at 54 °C (comp 65511 and 54981), or 58 °C (comp 57311, 24708, and EF1) for 1 min, and extension at 72 °C for 1 min.

### 3.8. Fluorescence Staining of the Sperm

AlexaFluor 488 was covalently bound to an amino group of AEBSF using the AlexaFluor 488 protein labeling kit (Molecular Probes, Eugene, OR, USA) according to the manufacturer’s instructions. To confirm the specific binding to serine or cysteine proteases, trypsin-, chymotrypsin- or papain-conjugated agarose beads (Sigma-Aldrich) were treated with 40 µg/mL AlexaFluor 488-labeled AEBSF in the RVDS for 15 min. After being washed with RVDS, the beads were observed with a fluorescence microscope (BH2-rfk; Olympus, Tokyo, Japan). For the fluorescence staining of the sperm with the AlexaFluor 488-labeled AEBSF, we pretreated sperm with 10 µM MitoTracker Orange (Molecular Probes, Eugene, OR, USA) in RVDS for 1 h and then treated with 40 µg/mL AlexaFluor 488-bound AEBSF and 0.2 µg/mL Hoechst33342 (Molecular Probes) in RVDS for 30 min. As the control, sperm were treated with RVDS or co-treated with 40 µg/mL AlexaFluor 488-labeled AEBSF and 1 mg/mL papain (Nacalai Tesque, Kyoto, Japan). They were rinsed with RVDS and observed with a confocal laser scanning microscope (C2; Nikon, Tokyo, Japan). Three-dimensional reconstruction of the fluorescence images was performed using the image analysis software Volocity (PerkinElmer, San Jose, CA, USA).

## 4. Conclusions

Sperm proteases are known to have multiple functions in fertilization depending on the animal species [17,18]. In the present study, we demonstrated that serine or cysteine protease in the acrosome and a papain-like cysteine protease in the principal piece may have a distinct key role in the initiation of sperm motility in *C. pyrrhogaster.* Although some proteases in seminal fluid is suggested to be a direct inducer for activation of sperm motility in arthropods [36], the participation of sperm proteases in the initiation of sperm motility is quite unique in the internal fertilization of *C. pyrrhogaster*. We also detected two acrosins and one 20S proteasome as possible candidates for acrosomal proteases. Further molecular identification of sperm proteases will contribute significantly to our understanding of how the sperm proteases act in the mechanism for the initiation of sperm motility.

## Supplementary Materials

Supplementary Table can be found at http://www.mdpi.com/1422-0067/15/9/15210/s1.

## Supplementary Files

Supplementary File 1
