# Supplementary material for: Sperm Proteases that May Be Involved in the Initiation of Sperm Motility in the Newt, Cynops pyrrhogaster
Source: Int J Mol Sci. 2014 Aug 28;15(9):15210–24. doi: 10.3390/ijms150915210 (PMC4200841; doi:10.3390/ijms150915210)
Supplement: Supplementary File 1 [file ijms-15-15210-s001.pdf]

Supplementary Information

Table S1. Base sequence of DNA primers for RT-PCR.

Contigs & Gene	Forward	Reverse
comp 65511	AAAGGAGAGGAAAGGAATGTTCA	GGCAACTGTCCACTCCACCCTTC
comp 57311	GTACTGACTGCAGCCCACTGCTT	CACAGGTTGTAGTCCTTGATAGC
comp 54981	CAGACCCTTTGGTATATCTGCTT	CTGTTTGTGTGCACGTTGCTTGC
comp 24708	ATATGCCATGGAAGCAGTCAAGC	ACGAGCTCCTATAGACATGGCTT
EF1	AGGTATTGGCACAGTCCCAGTGG	TCAAGCTTCTTGCCAGATCGTCTG
